# Infection or contamination – Clinical attribution of blood culture-positive episodes in neonatal intensive care: an 11-year retrospective single-center study

**DOI:** 10.1186/s12879-026-14605-8

**Published:** 2026-10-15

**Authors:** Sarah V. Schnee, Florens Lohrmann, Daniel Matheisl, Hendryk Schneider, Markus Hufnagel, Hans Fuchs, Martin Kuntz

**Affiliations:** 1 Division of Neonatology and Pediatric Critical Care Medicine, Department of General Pediatrics, Adolescent Medicine and Neonatology, Center for Pediatrics, Medical Center and Faculty of Medicine, University of Freiburg, Freiburg, Germany; 2 Division of Pediatric Rheumatology and Clinical Infectious Diseases, Department of General Pediatrics, Adolescent Medicine and Neonatology, Center for Pediatrics, Medical Center and Faculty of Medicine, University of Freiburg, Freiburg, Germany

**Keywords:** Neonatal sepsis, Bloodstream infection, Contamination, Epidemiology

## Abstract

**Background:**

Positive blood cultures in neonatal intensive care do not necessarily indicate clinically relevant bloodstream infection. Interpretation is particularly challenging for coagulase-negative staphylococci (CoNS), which may represent either contamination or clinically relevant infection depending on the clinical context. We aimed to describe how treating teams attributed culture-positive episodes as infection or contamination in routine neonatal care and to examine which clinical, laboratory, and microbiological features were associated with this original clinical attribution. As a secondary aim, we characterized the local epidemiology of culture-positive episodes.

**Methods:**

We conducted a retrospective single-center study of all culture-positive episodes occurring between 2014 and 2024 in neonates hospitalized in a German tertiary NICU at the time of blood culture sampling. Original clinical attribution was extracted from documentation recorded during the index episode. Descriptive analyses and exploratory logistic regression were performed.

**Results:**

Among 3246 blood cultures, 232 (7.1%) were positive. After accounting for additional follow-up and parallel cultures and other exclusions, 189 unique culture-positive episodes remained for analysis; 125 (66.1%) were attributed as infection and 64 (33.9%) as contamination. Thirty-five episodes (18.5%) occurred during the early-onset period and 154 (81.5%) during the late-onset period. Coagulase-negative staphylococci (CoNS) were detected in 114 episodes (60.3%) and showed the greatest variability in attribution. All 13 CoNS-positive early-onset episodes were attributed as contamination, whereas 64 of 101 CoNS-positive late-onset episodes (63.4%) were attributed as infection. Enterobacterales, *Enterococcus faecalis*, *Staphylococcus aureus*, and *Candida* spp. were almost uniformly attributed as infection, whereas *Bacillus* spp. and environmental organisms were predominantly attributed as contamination. In late-onset episodes, maximum CRP showed the strongest association with attribution as infection in univariable and multivariable analyses (adjusted OR 4.41, 95% CI 2.59–7.51). Associations with time-to-positivity, CoNS detection, and central vascular catheter presence were weaker and imprecisely estimated after adjustment.

**Conclusions:**

Clinical attribution of culture-positive episodes was associated with the combined consideration of organism plausibility, inflammatory response, time-to-positivity, and clinical context. CoNS-positive late-onset episodes represented the greatest diagnostic uncertainty. These findings describe real-world clinical attribution rather than diagnostic accuracy and underline the need for more reproducible criteria to support antimicrobial stewardship without compromising the treatment of vulnerable neonates.

**Supplementary Information:**

The online version contains supplementary material available at https://doi.org/10.1186/s12879-026-14605-8.

## Introduction

Neonatal sepsis remains a major cause of morbidity and mortality, particularly among critically ill and very preterm infants in neonatal intensive care units (NICU) and require rapid recognition and timely antimicrobial treatment. Blood cultures are central to the diagnosis of bloodstream infection, yet culture positivity does not necessarily indicate clinically relevant infection. In neonates, early clinical signs of infection are often subtle and nonspecific and may overlap with non-infectious conditions. In addition, limited vascular access and difficulties in obtaining sufficient blood samples complicate microbiological assessment, while contamination may produce ambiguous positive results [1–3].

The epidemiology of neonatal bloodstream infection is influenced by gestational age, postnatal age, duration of hospitalization, and exposure to invasive interventions. In typical high-income NICU contexts, the organism spectrum differs between early- and late-onset infection. Early-onset infection is typically associated with vertically transmitted organisms, mainly group B streptococci and *Escherichia coli*, with *E. coli* being particularly relevant among preterm infants. In contrast, late-onset infection is more strongly influenced by postnatal intensive care exposure and local unit microbiological ecology and is frequently associated with healthcare-associated organisms such as coagulase-negative staphylococci (CoNS), *Staphylococcus aureus*, and Gram-negative bacteria. Although this general distinction is consistent across cohorts, the precise distribution of late-onset organisms varies between NICU, likely reflecting differences in patient populations, device exposure, antimicrobial practices, and local microbiological ecology [1, 4–7].

These epidemiological differences are clinically relevant for two reasons. On the one hand, they are the basis for the rational choice of empiric antibiotic regimens. On the other hand, they affect the plausibility of a positive blood culture to represent true invasive infection. This is especially evident for CoNS-positive episodes. As common skin commensals, CoNS are frequent contaminants but are also established causes of catheter-associated and biofilm-related bloodstream infection in preterm infants with prolonged hospitalization and intravascular devices. Their interpretation is therefore especially challenging in the late-onset period, where they are frequently detected, whereas during the early-onset period they are generally less plausible as vertically acquired invasive pathogens [8, 9].

This diagnostic uncertainty is further complicated by the absence of uniform neonatal sepsis definitions. Surveillance systems such as the German NeoKISS protocol provide standardized criteria for epidemiological surveillance and quality assurance but are not intended to replace individualized clinical bedside judgement [10]. At the same time, antimicrobial therapy is often initiated empirically, making the interpretation of positive blood cultures therefore critical to decisions on continuation, modification, or discontinuation of therapy within antimicrobial stewardship framework.

In this study, first, we aimed to describe the epidemiology of culture-positive episodes by onset period and organism group in a German tertiary care NICU – and second, we aimed to describe how treating teams attributed culture-positive episodes as infection or contamination and to examine which clinical, laboratory, and microbiological features were associated with the original clinical attribution, with particular emphasis on CoNS.

## Methods

### Study design and population

This retrospective single-center study was conducted at the NICU of a German tertiary care university hospital. All culture-positive episodes occurring from 2014 to 2024 in neonates who were hospitalized in the NICU at the time of blood culture sampling were reviewed. Neonates presenting from home through the emergency department with community-onset late infection were not included. Demographic, clinical, laboratory, and microbiological data were retrieved from electronic medical records and discharge summaries.

### Episode and variable definitions

A culture-positive episode was defined as one or more positive blood cultures obtained as part of the same diagnostic work-up and attributed to the same clinical event. Additional positive blood cultures obtained simultaneously or during the same clinical event were combined into a single episode to avoid duplicate counting. Parallel cultures were defined as additional blood cultures obtained at the same sampling time. Follow-up cultures were defined as repeated positive cultures yielding the same species within one week of the index culture when a temporal and clinical relationship to the index episode was evident. Cultures representing a distinct clinical event, including recurrent episodes in the same infant, were counted as separate episodes. Polymicrobial episodes were defined as episodes in which more than one organism was detected within the same diagnostic event.

Episodes were assigned to the early-onset period when the index blood culture was obtained within the first 72 h of life and to the late-onset period when sampling occurred after 72 h of life. These categories describe the timing of blood culture sampling only and do not imply that the episode represented sepsis or bloodstream infection.

CRP at sampling was defined as the value obtained closest to blood culture collection within 2 h. Maximum CRP was defined as the highest value documented within the same episode (maximum one week after blood culture date). Time-to-positivity (TTP) was defined as the interval between initiation of blood culture incubation and automated detection of microbial growth. Central vascular catheter presence was assessed at the time of sampling. Postoperative status was defined as surgery within the same hospital stay.

### Extraction of the original clinical attribution

The primary outcome was the original clinical attribution of each culture-positive episode as infection or contamination by the treating team during the index episode. The treating team’s assessment was extracted from the medical file of the patient. Discharge summaries, daily progress notes, microbiology-related documentation and other medical records from the original treatment period were reviewed by one experienced neonatologist for an explicit statement regarding the clinical interpretation of the positive blood culture. In case no explicit assessment was documented, or if documentation was ambiguous, the treating team’s original interpretation was reconstructed from subsequent management decisions, particularly whether antimicrobial therapy was discontinued, continued, prolonged, or modified after receiving the blood culture result. There was no attempt to retrospectively verify the plausibility of the original assessment. Throughout the manuscript, attribution as infection or contamination therefore refers to the original assessment made by the treating team.

### Blood culture sampling and microbiology

Blood culture sampling was standard procedure before initiation or modification of antimicrobial therapy in our unit. Skin antisepsis was performed using 0.1% octenidine dihydrochloride in preterm infants weighing < 1500 g and an alcohol-based antiseptic in all other infants. The recommended blood culture volume was at least 0.5 mL in preterm infants and 1 mL in term infants; actual inoculated volumes were not routinely recorded. BacT/ALERT PF pediatric blood culture bottles (bioMérieux Deutschland, Nürtingen, Germany) were used throughout the study period. Following collection, blood culture bottles were transferred for incubation and automated microbial growth detection according to the routine microbiological workflow.

For blood cultures obtained outside routine opening hours of the microbiology laboratory, bottles underwent pre-incubation in the central laboratory or, during earlier parts of the study period, within the children’s hospital before transfer to the microbiology laboratory. Time-to-positivity could not be determined for pre-incubated cultures. All 17 missing TTP values in the late-onset cohort were attributable to pre-incubation.

The sampling site was documented for 116 of 189 culture-positive episodes (61.4%): 88 were obtained peripherally and 28 from a central vascular catheter. Because of the substantial proportion of missing data, sampling site was not included as an analytical variable.

Routine surveillance cultures were obtained according to local infection-control screening practices and did not systematically include CoNS. Consequently, ascertainment of concordant organism detection in surveillance cultures differed across organism groups.

### Organism classification

Detected pathogens were grouped into clinically relevant categories. Enterobacterales included *Escherichia coli*, *Klebsiella* spp., *Enterobacter cloacae* complex, and *Serratia marcescens*. Coagulase-negative staphylococci (CoNS) comprised *S. epidermidis*, *S. capitis*, *S. haemolyticus*, *S. hominis*, *S. warneri*, and *S. auricularis*. Other groups included *Staphylococcus aureus*, *Enterococcus faecalis*, *Streptococcus* spp., *Bacillus* spp., anaerobes such as *Cutibacterium acnes*, environmental bacteria such as *Micrococcus* spp. and *Moraxella*, opportunistic pathogens such as *Stenotrophomonas maltophilia*, and yeasts such as *Candida* spp.

### Statistical analysis

Continuous variables are presented as mean and standard deviation or median and interquartile range, as appropriate. Categorical variables are presented as counts and percentages. The number of available observations varied between analyses because not all laboratory parameters were available for every episode. IL-6 was available only from year 2021 onwards.

Univariable logistic regression was used to examine which clinical, laboratory, and microbiological features were associated with original clinical attribution as infection. The binary outcome was coded as 1 for attribution as infection and 0 for attribution as contamination. Maximum CRP was transformed using ln(CRP + 1). Time-to-positivity was modeled per one-hour increase, gestational age and postnatal age per one-week increase.

Regression analyses were restricted to late-onset culture-positive episodes because early- and late-onset episodes represent distinct clinical and microbiological settings, the number of early-onset episodes was limited, and all CoNS-positive early-onset episodes were attributed as contamination. Multivariable analyses were based on complete cases. Because some infants contributed more than one late-onset episode, the multivariable analysis was additionally repeated using cluster-robust standard errors at the infant level. Odds ratios are presented with 95% Wald confidence intervals. Statistical significance was assessed using two-sided Wald tests, with *p* < 0.05 considered statistically significant.

All analyses and visualizations were performed using R version 4.4.3 (R Foundation for Statistical Computing, Vienna, Austria).

### Ethics

The study was conducted in accordance with the revised Declaration of Helsinki of the World Medical Association, applicable federal and state laws, and the professional code of conduct for physicians. It was approved by the Ethics Committee of the University Medical Center Freiburg (reference 22-1289). The requirement for informed consent was waived because of the retrospective study design.

## Results

### Cohort and episode characteristics

Between 2014 and 2024, 3246 blood cultures were obtained from 2268 neonates admitted to the NICU, of which 232 (7.1%) were positive.

Of 232 positive blood cultures, 43 were not included as separate culture-positive episodes, leaving 189 episodes for analysis. Of these 43 cultures, 41 represented additional positive cultures belonging to 30 clinical episodes: 32 were follow-up cultures and 9 were parallel cultures. In all 43 cultures the same species was recovered as in the index culture. All 30 corresponding episodes were originally attributed as infection. Two further positive cultures were excluded: in one case, attribution as infection or contamination could not be reliably reconstructed from the clinical record; the other represented an exceptional iatrogenic event.

**Fig. 1:**
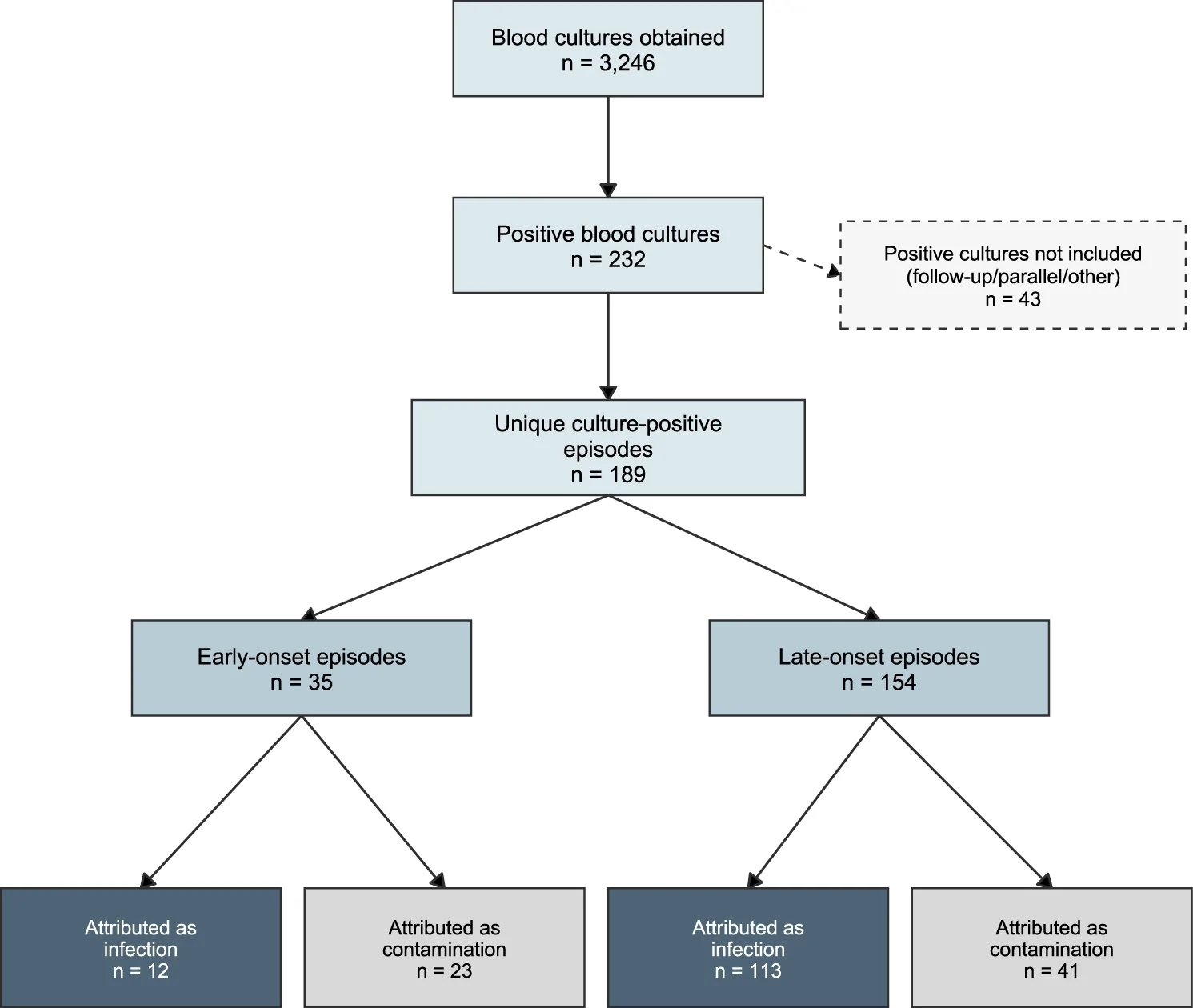
Flow diagram of blood cultures and culture-positive episodes included in the study. 189 culture-positive episodes included. 43 positive cultures not included as separate episodes due to additional follow-up or parallel cultures or other exclusions. Episodes were subsequently stratified by onset period and original clinical attribution

Thirty-five episodes (18.5%) occurred during the early-onset period and 154 (81.5%) during the late-onset period. Overall, 125 episodes (66.1%) were attributed as infection and 64 (33.9%) as contamination (Fig. 1). Clinical attribution of positive blood cultures was readily apparent from the original documentation in all included cases per the criteria described above.

Relative to all blood cultures obtained during the study period, this corresponded to 71.5 positive cultures and 58.2 unique culture-positive episodes per 1000 blood cultures. Of these, 38.5 episodes were attributed as infection and 19.7 as contamination per 1000 blood cultures.

**Fig. 2:**
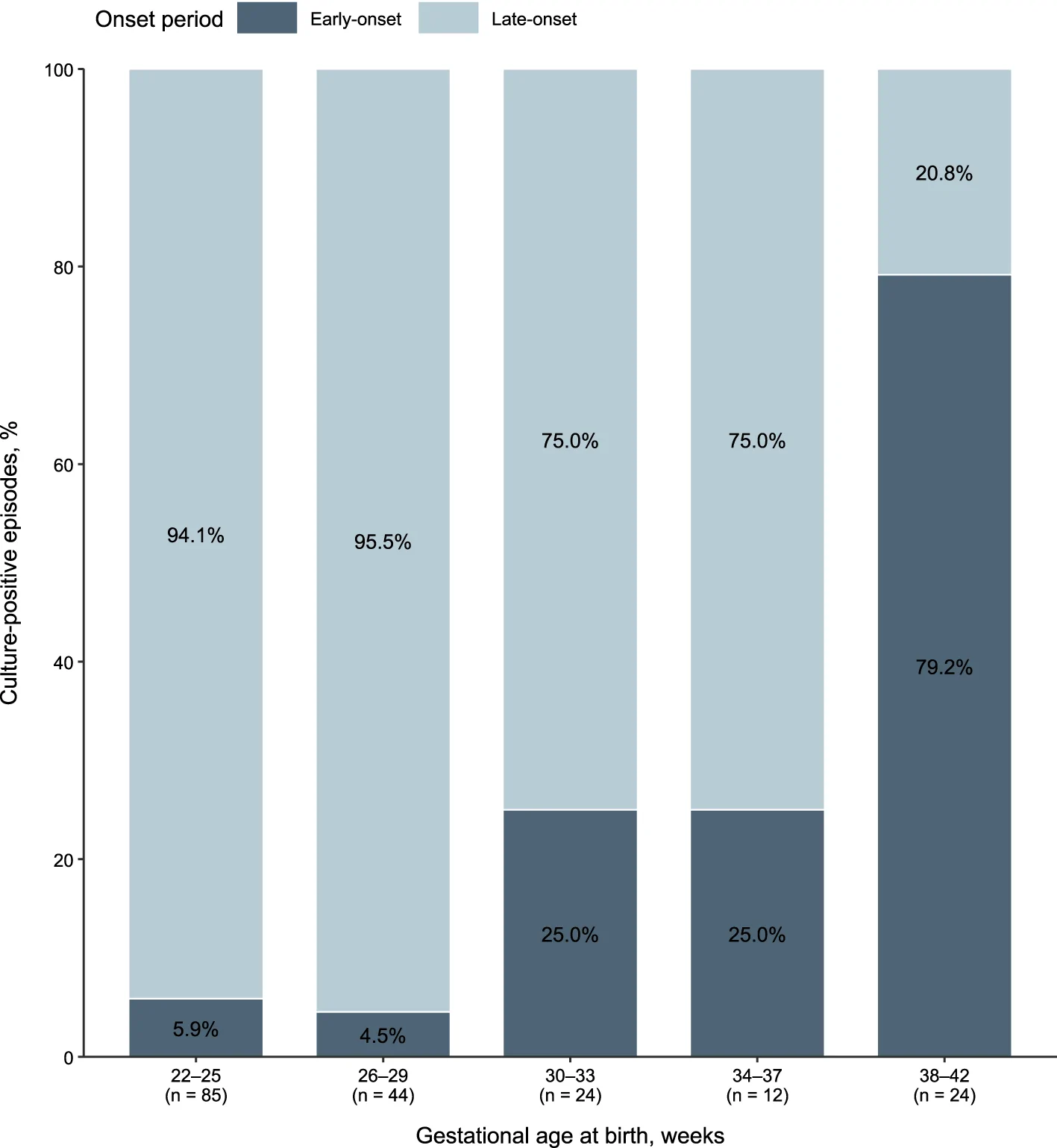
Distribution of early-onset and late-onset culture-positive episodes by gestational age group. Bars show the proportion of culture-positive episodes assigned to the early-onset or late-onset period within each gestational age group. Percentages are calculated within gestational age groups

The mean gestational age at birth was 28.3 ± 5.6 weeks, with a range of 22–42 weeks. The distribution of onset periods differed across gestational age groups (Fig. 2). Among infants born before 30 weeks’ gestation, 94.0–96.0% of culture-positive episodes occurred during the late-onset period. Early-onset episodes accounted for approximately one quarter of episodes among infants born between 30 and 37 weeks and 79.0% of episodes among term infants.

**Fig. 3:**
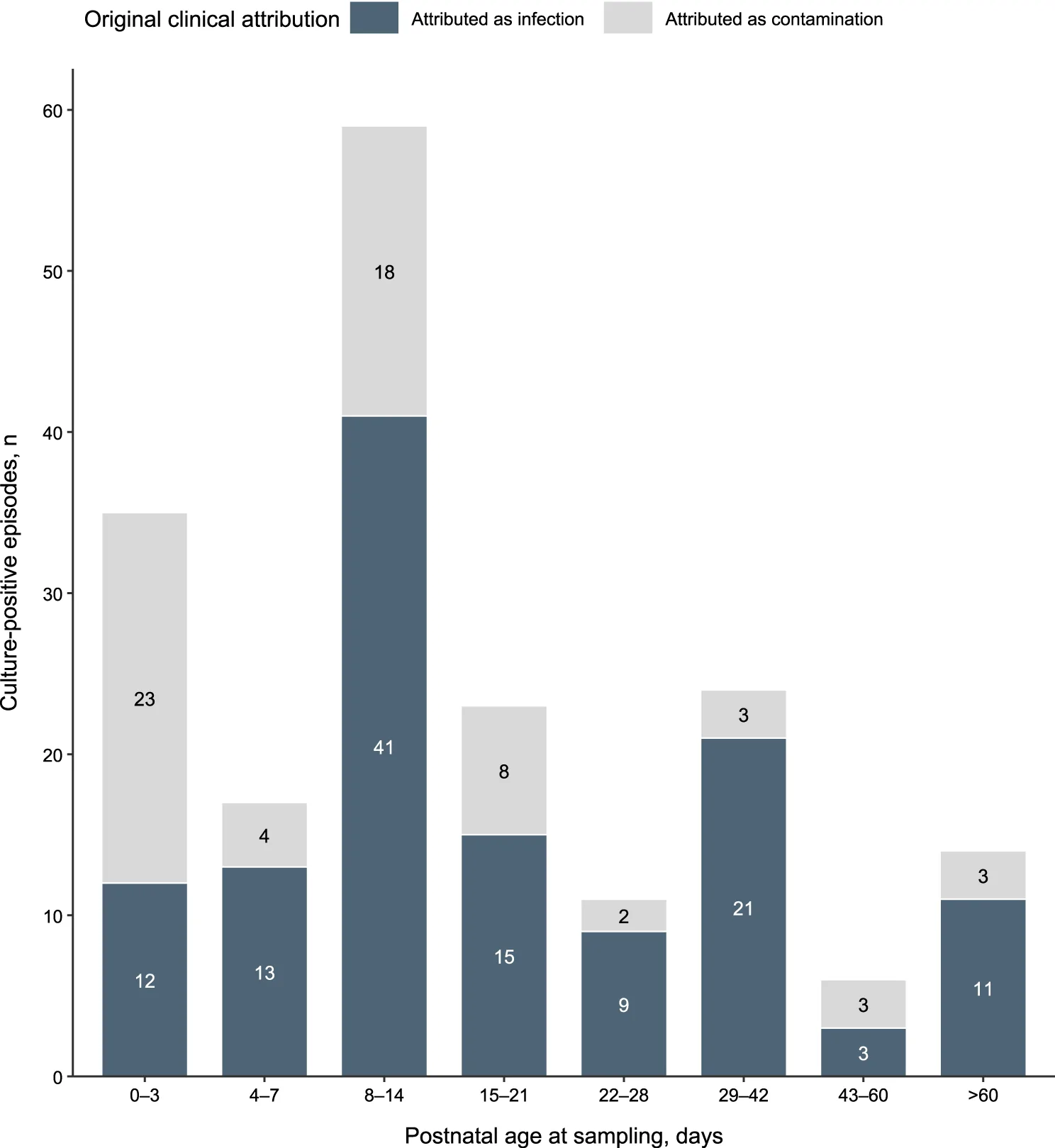
Distribution of culture-positive episodes by postnatal age at blood culture sampling and original clinical attribution. Bars show the absolute number of episodes within predefined postnatal age intervals, stratified by attribution as infection or contamination

Culture-positive episodes clustered during the first postnatal days and around the second postnatal week, reflecting the different timing of early-onset and hospital-associated late-onset episodes (Fig. 3). The early peak contained a high proportion of episodes attributed as contamination, whereas episodes attributed as infection peaked around postnatal day 10.

### Pathogen distribution by onset period

Across the 189 culture-positive episodes, 31 different pathogens were identified. CoNS were the most frequently detected group, accounting for 114 episodes (60.3%), followed by Enterobacterales in 33 (17.5%), *Streptococcus* spp. and *Enterococcus faecalis* in 10 each (5.3%), *Bacillus* spp. in seven (3.7%), environmental organisms in six (3.2%), and *Staphylococcus aureus* in four (2.1%). Multidrug-resistant organisms were rare, with two episodes involving 3MRGN *E. coli*, including one ESBL-producing isolate. Pathogen groups by onset period and original clinical attribution are shown in Table 1. The species-level distribution of all organism detections, including additional organisms identified in polymicrobial episodes, is provided in Supplementary Table S1.

Among the 35 early-onset episodes, detected organisms included CoNS in 13 (37.1%), streptococci in eight (22.9%), Enterobacterales in five (14.3%), *Bacillus* spp. in three (8.6%), and other organisms in six (17.1%). Episodes attributed as infection were mainly caused by streptococci and Enterobacterales. Four GBS-positive episodes were identified, all in term infants. Intrapartum antibiotic prophylaxis had not been administered or maternal GBS status was unknown.

The late-onset pathogen spectrum differed markedly. CoNS were detected in 101 of 154 episodes (65.6%), followed by Enterobacterales in 28 (18.2%) and *E. faecalis* in nine (5.8%). *S. aureus* and other organisms were less frequent. Among late-onset episodes with detection of the same organism in surveillance cultures, the most frequently identified organisms were *E. coli*, *E. faecalis*, *E. cloacae* complex, and *K. pneumoniae*.

Clinical attribution varied substantially by pathogen group. *Candida* spp., Enterobacterales, *E. faecalis*, and *S. aureus* were almost uniformly attributed as infection, whereas *Bacillus* spp. and environmental organisms were predominantly attributed as contamination (Table 1).

**Table 1:** Pathogen groups detected in culture-positive episodes by onset period and original clinical attribution. Values are presented as n (column %). Each episode was assigned to one organism group for this episode-level analysis; additional organisms detected in polymicrobial episodes are listed in Supplementary Table S1

Organism group	Overall (*n* = 189)	Early-onset: attributed as infection (*n* = 12)	Early-onset: attributed as contamination (*n* = 23)	Late-onset: attributed as infection (*n* = 113)	Late-onset: attributed as contamination (*n* = 41)
Coagulase-negative staphylococci	114 (60.3)	0 (0.0)	13 (56.5)	64 (56.6)	37 (90.2)
Enterobacterales	33 (17.5)	5 (41.7)	0 (0.0)	28 (24.8)	0 (0.0)
*Streptococcus* spp.	10 (5.3)	5 (41.7)	3 (13.0)	2 (1.8)	0 (0.0)
*Enterococcus faecalis*	10 (5.3)	0 (0.0)	0 (0.0)	9 (8.0)	1 (2.4)
*Bacillus* spp.	7 (3.7)	2 (16.7)	1 (4.3)	2 (1.8)	2 (4.9)
Environmental organisms	6 (3.2)	0 (0.0)	5 (21.7)	0 (0.0)	1 (2.4)
*Staphylococcus aureus*	4 (2.1)	0 (0.0)	0 (0.0)	4 (3.5)	0 (0.0)
Yeasts	2 (1.1)	0 (0.0)	0 (0.0)	2 (1.8)	0 (0.0)
Anaerobes	2 (1.1)	0 (0.0)	1 (4.3)	1 (0.9)	0 (0.0)
Opportunistic pathogens	1 (0.5)	0 (0.0)	0 (0.0)	1 (0.9)	0 (0.0)

**Fig. 4:**
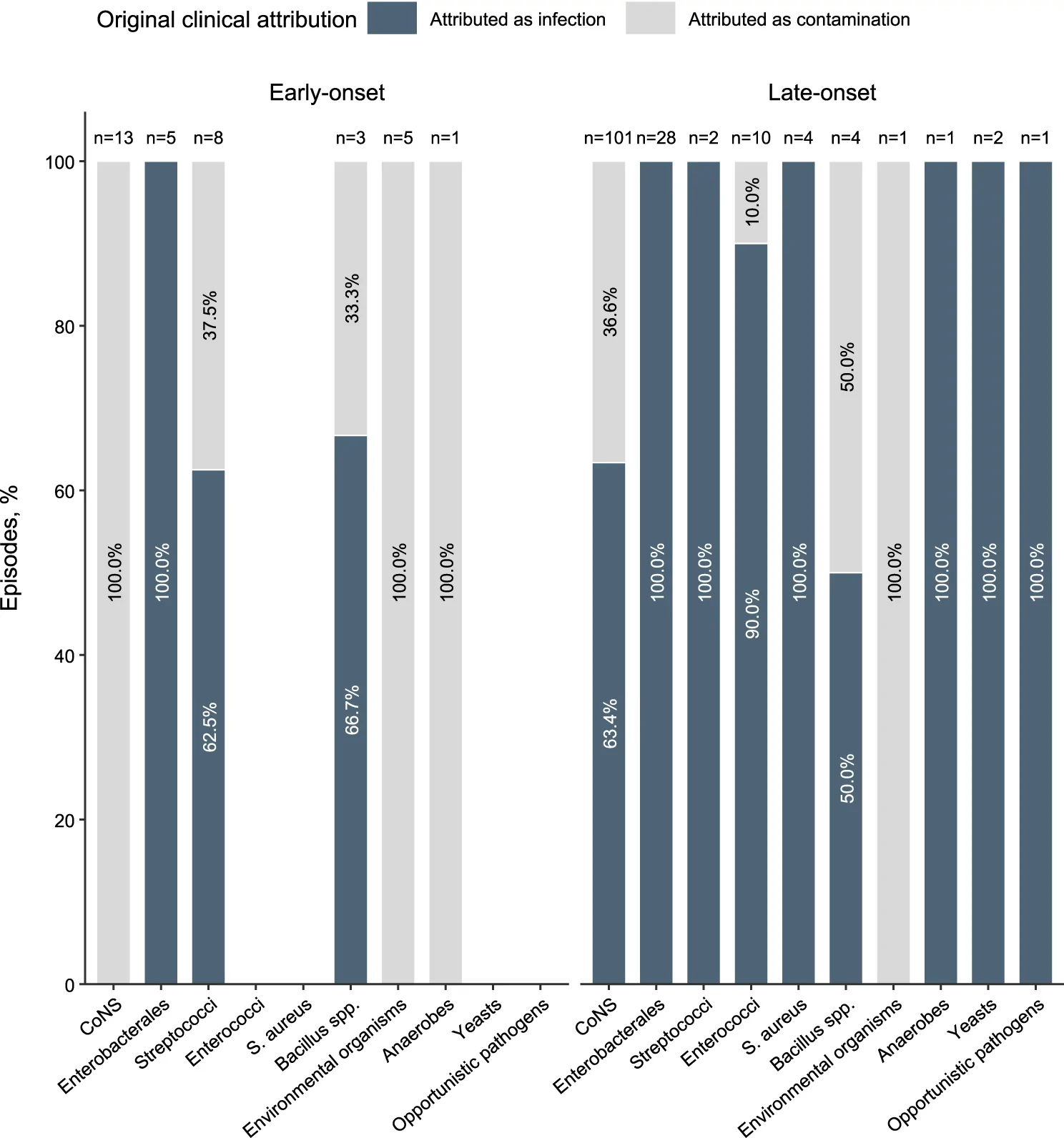
Distribution of selected pathogen groups by onset period and original clinical attribution. Bars show the proportion of episodes attributed as infection or contamination within each pathogen group. Numbers above the bars indicate the total number of episodes in each pathogen group

CoNS showed the greatest variability in original clinical attribution. All 13 CoNS-positive early-onset episodes were attributed as contamination, whereas among 101 CoNS-positive late-onset episodes, 64 (63.4%) were attributed as infection and 37 (36.6%) as contamination (Fig. 4).

Polymicrobial detections occurred in 26 episodes (13.8%) and most frequently comprised combinations of different CoNS species. 23 of 26 polymicrobial episodes (88.5%) occurred during the late-onset period, 24 (92.3%) affected preterm infants born between 22 and 33-weeks’ gestation, and 18 (69.2%) occurred in the presence of a central vascular catheter. Sixteen episodes (61.5%) were attributed as infection; all occurred during the late-onset period, whereas the three polymicrobial early-onset episodes were attributed as contamination.

### Early-onset culture-positive episodes

The 35 early-onset episodes occurred predominantly in term infants, with a median gestational age of 39 weeks and a median postnatal age of 1 day at sampling. A central vascular catheter was present in three episodes (8.6%), no episode occurred after surgery, and maternal colonization was documented in six episodes (17.1%).

Of the 35 episodes, 12 (34.3%) were attributed as infection and 23 (65.7%) as contamination. CRP concentrations at blood culture sampling were generally low and showed little separation between attribution groups, whereas maximum CRP was higher in episodes attributed as infection than in those attributed as contamination (median 44 vs. 13 mg/L). IL-6 was available in 26 episodes and was markedly higher at sampling in episodes attributed as infection (median 6042 vs. 98 pg/mL). Time-to-positivity was also shorter in episodes attributed as infection (median 8 vs. 22 h). Detailed characteristics are provided in Supplementary Table S2. Because of the limited number of episodes and the distinct perinatal setting, early-onset episodes were analyzed descriptively.

### Late-onset culture-positive episodes

The 154 late-onset episodes occurred in 132 infants, predominantly very preterm with a median gestational age of 25 weeks and a median postnatal age of 15 days at sampling. A central vascular catheter was present in 122 episodes (79.2%), 30 episodes (19.5%) occurred after surgery, and detection of the same organism in surveillance cultures was documented in 44 episodes (28.6%). In 71.0% of these episodes, the organism had been documented before blood culture sampling; in the remaining episodes, first detection in surveillance and blood cultures occurred during the same diagnostic period.

Of the 154 episodes, 113 (73.4%) were attributed as infection and 41 (26.6%) as contamination. Gestational age, postnatal age at sampling, central vascular catheter exposure, and prior surgery showed little descriptive difference between attribution groups. CoNS detection was less frequent in episodes attributed as infection than in those attributed as contamination (56.6% vs. 90.2%). Episodes attributed as infection had higher CRP concentrations at sampling (median 23 vs. 0 mg/L) and higher maximum CRP values (54 vs. 0 mg/L), while time-to-positivity was shorter (12 vs. 18 h; Table 2).

**Table 2:** Characteristics of late-onset culture-positive episodes stratified by original clinical attribution. Values are presented as median (interquartile range) or n (%). Percentages were calculated within each column using episodes with available data as the denominator. No statistical comparisons were performed in this table; associations with clinical attribution are reported in Table 3. Data on CRP at sampling were available for 113 infection-attributed and 38 contamination-attributed episodes; maximum CRP for 112 and 39 episodes, respectively; and time-to-positivity for 97 and 40 episodes, respectively. CoNS, coagulase-negative staphylococci; CRP, C-reactive protein; TTP, time-to-positivity

Feature	Overall (*n* = 154)	Attributed as infection (*n* = 113)	Attributed as contamination (*n* = 41)	Missing
*Demographic and clinical characteristics*				
Gestational age at birth, weeks	25 (24–28)	25 (24–29)	25 (24–27)	0
Postnatal age at sampling, days	15 (10–31)	15 (9–31)	14 (11–24)	0
Central vascular catheter present, n (%)	122 (79.2)	90 (79.6)	32 (78.0)	0
Prior surgery, n (%)	30 (19.5)	23 (20.4)	7 (17.1)	0
*Microbiological characteristics*				
Colonization with same organism, n (%)	44 (28.6)	42 (37.2)	2 (4.9)	0
CoNS detection, n (%)	101 (65.6)	64 (56.6)	37 (90.2)	0
*Inflammatory markers and time-to-positivity*				
CRP at sampling, mg/L	12 (1-36.5)	23 (9–50)	0 (0–4)	3
Maximum CRP, mg/L	35 (7.5–86.5)	54 (28–110)	0 (0-5.5)	3
TTP, h	15 (9–19)	12 (8–18)	18 (10.8–23.2)	17

**Fig. 5:**
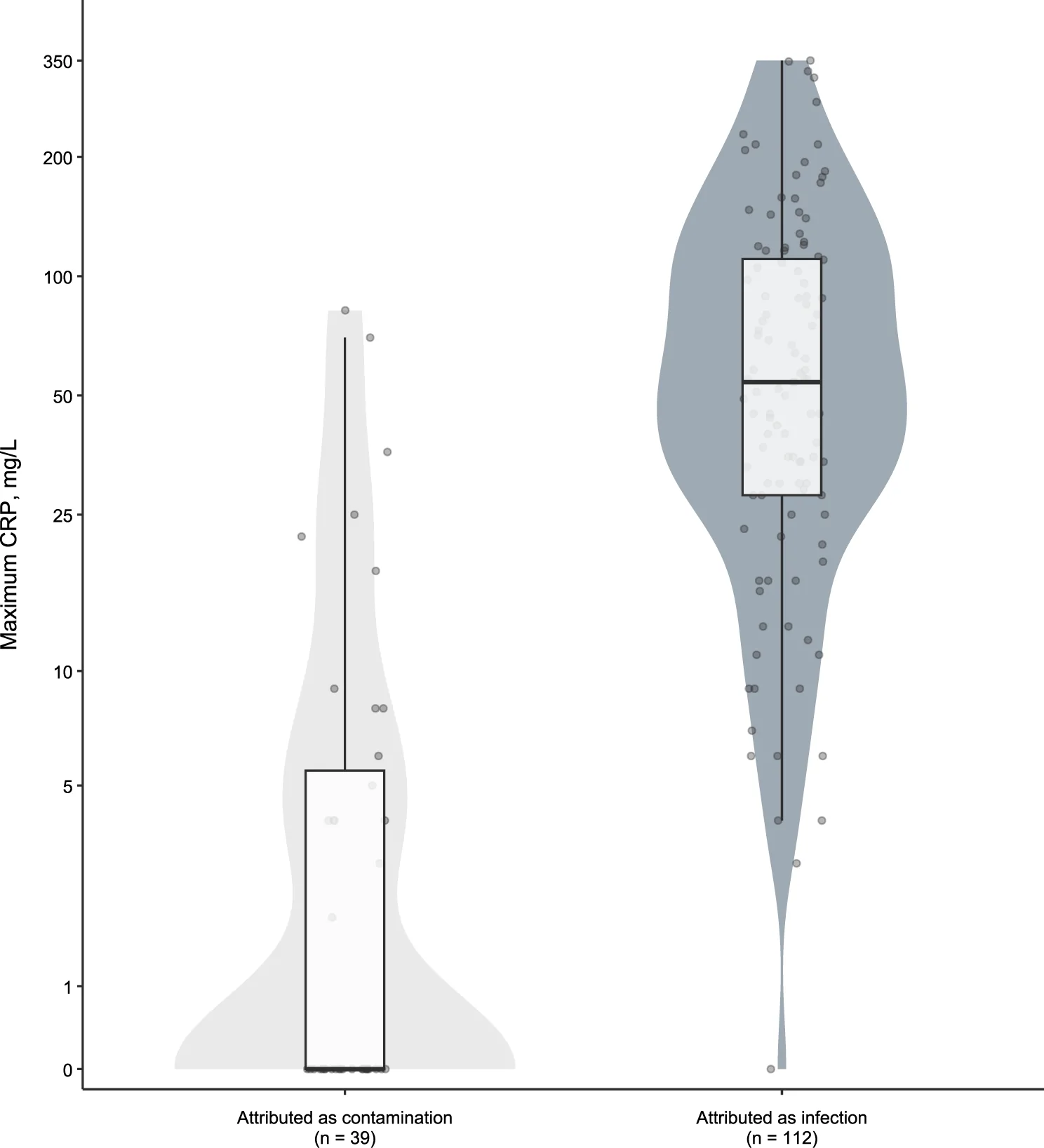
Distribution of maximum CRP concentrations in late-onset culture-positive episodes stratified by original clinical attribution. Violin plots show the distribution of maximum CRP concentrations, boxes indicate the interquartile range and median, and points represent individual episodes. The y-axis uses a pseudologarithmic scale to allow visualization of zero values. Three episodes with missing maximum CRP values were omitted

Despite these overall differences, the CRP distributions overlapped between attribution groups (Fig. 5). Eleven late-onset episodes were attributed as infection despite CRP concentrations below 10 mg/L both at sampling and during the subsequent observation period. Most of these episodes (*n* = 9, 81.8%) were CoNS-positive. Conversely, six episodes attributed as contamination had maximum CRP concentrations above 10 mg/L.

### Univariable associations with clinical attribution

We subsequently examined which clinical, laboratory, and microbiological features were associated with original clinical attribution as infection. In univariable logistic regression, higher maximum CRP showed the strongest association with attribution as infection (OR 4.39, 95% CI 2.75–7.03). Longer time-to-positivity (OR 0.98 per hour, 95% CI 0.96–1.00) and CoNS detection (OR 0.14, 95% CI 0.05–0.42) were associated with lower odds. Gestational age, postnatal age at sampling, central vascular catheter presence, and prior surgery showed no clear association (Table 3).

Because surveillance-culture ascertainment was not standardized across organism groups, particularly for CoNS, this variable was retained for descriptive analyses only and was not included in the univariable or multivariable regression analyses.

### Multivariable associations with clinical attribution

The exploratory multivariable model was intentionally restricted to a small set of clinically relevant variables representing different domains of clinical assessment: inflammatory response (maximum CRP), microbial growth dynamics (time-to-positivity), organism context (CoNS), and device-related clinical context (central vascular catheter presence). Variable selection was informed by clinical relevance, measurement quality, and the univariable findings, but was not based on a formal significance threshold. Postoperative status was evaluated in univariable analysis but was not included in the multivariable model because postoperative inflammation may increase CRP independently of infection.

The final multivariable model included log-transformed maximum CRP, time-to-positivity, CoNS detection, and central vascular catheter presence and was based on 134 episodes with complete data. Maximum CRP remained strongly associated with attribution as infection (adjusted OR 4.41, 95% CI 2.59–7.51). Associations with time-to-positivity (adjusted OR 0.98 per hour, 95% CI 0.95–1.00), CoNS detection (adjusted OR 0.54, 95% CI 0.12–2.43), and central vascular catheter presence (adjusted OR 3.25, 95% CI 0.64–16.41) were weaker and imprecisely estimated (Table 3).

Replacing maximum CRP with CRP measured at blood-culture sampling, CRP at sampling remained strongly associated with attribution as infection (aOR 2.78, 95% CI 1.90–4.06; *p* < 0.001; *n* = 133).

Excluding TTP from the multivariable model increased the analyzable sample from 134 to 151 episodes and did not materially alter the association between maximum CRP and original clinical attribution as infection (aOR 4.37, 95% CI 2.65–7.20; *p* < 0.001).

The multivariable complete-case analysis included 134 late-onset episodes from 116 infants; 15 infants contributed more than one episode. A sensitivity analysis using infant-level cluster-robust standard errors yielded materially unchanged results, maximum CRP remained strongly associated with attribution as infection (aOR 4.41, 95% CI 2.76–7.04; *p* < 0.001).

**Table 3:** Univariable and multivariable logistic regression analysis of clinical, laboratory, and microbiological features associated with original clinical attribution as infection among late-onset culture-positive episodes. Models were restricted to late-onset episodes. The multivariable model was based on complete cases (*n* = 134). Odds ratios > 1 indicate higher odds of clinical attribution as infection. Maximum CRP was log-transformed because of right-skewed distribution, so the corresponding odds ratio refers to a one-unit increase in the log-transformed value and not to an increase of 1 mg/L. TTP was modeled per one-hour increase. Postnatal age at sampling was modeled per 7-day increase. CoNS detection was compared with all non-CoNS organisms

Feature	Unadjusted OR (95% CI)	*p* value	Adjusted OR (95% CI)	*p* value
Maximum CRP, log-transformed	4.39 (2.75–7.03)	< 0.001	4.41 (2.59–7.51)	< 0.001
TTP, per hour	0.98 (0.96–1.00)	0.042	0.98 (0.95-1.00)	0.095
CoNS detection	0.14 (0.05–0.42)	< 0.001	0.54 (0.12–2.43)	0.424
Central vascular catheter present	1.10 (0.46–2.63)	0.829	3.25 (0.64–16.41)	0.154
Prior surgery	1.24 (0.49–3.16)	0.650	-	-
Gestational age at birth, per week	0.98 (0.90–1.07)	0.705	-	-
Postnatal age at sampling, per 7-day increase	1.01 (0.92–1.12)	0.840	-	-

### CoNS-positive late-onset subgroup

Among 101 CoNS-positive late-onset episodes, 64 (63.4%) were attributed as infection and 37 (36.6%) as contamination. Episodes attributed as infection had higher CRP concentrations at sampling (median 13 vs. 0 mg/L) and higher maximum CRP values (40 vs. 0 mg/L), while time-to-positivity was moderately shorter (15 vs. 18.5 h). Gestational age, postnatal age, central vascular catheter exposure, and prior surgery showed little descriptive difference between groups. Detailed characteristics are provided in Supplementary Table S3.

In univariable analyses restricted to CoNS-positive late-onset episodes, higher maximum CRP remained strongly associated with original clinical attribution as infection (OR 3.92, 95% CI 2.38–6.43; *p* < 0.001; *n* = 98). Associations with time-to-positivity (OR 0.98 per hour, 95% CI 0.96–1.00; *p* = 0.103; *n* = 94) and central vascular catheter presence (OR 1.49, 95% CI 0.53–4.19; *p* = 0.450; *n* = 101) were weaker.

In a multivariable analysis restricted to CoNS-positive late-onset episodes, maximum CRP remained strongly associated with original clinical attribution as infection (aOR 4.46, 95% CI 2.47–8.06; *p* < 0.001). Shorter time-to-positivity was also associated with attribution as infection (aOR 0.97 per hour, 95% CI 0.94–1.00; *p* = 0.038), whereas the estimate for central vascular catheter presence was imprecise (aOR 6.31, 95% CI 0.97–40.92; *p* = 0.053).

### Clinical management and outcomes

Empirical antibiotic therapy was initiated at the time of blood culture sampling in most episodes. In 15 late-onset episodes, treatment was started later, most commonly within 10–24 h, in the context of initially subtle clinical presentations and low CRP concentrations.

Nine infants died during hospitalization, including two with early-onset and seven with late-onset episodes. Most were extremely preterm infants with severe underlying comorbidities. In two cases, death occurred in close temporal association with the culture-positive episode. Given the small number of deaths and the high burden of underlying morbidity, no formal attribution or outcome analysis was performed.

## Discussion

This retrospective study of culture-positive episodes in a German tertiary NICU highlights a central challenge in neonatal infectious disease diagnostics: culture positivity is not equivalent to clinically relevant bloodstream infection. We therefore examined the original clinical attribution of each episode as infection or contamination. This distinction is relevant to antimicrobial stewardship, because over-attribution may lead to unnecessary continuation of antibiotic therapy, whereas under-attribution may result in insufficient treatment of clinically relevant infection. Surveillance frameworks such as NeoKISS acknowledge this uncertainty, particularly for CoNS-positive episodes [10, 11], but are designed primarily for standardized epidemiological surveillance and are not equivalent to bedside attribution. A formal concordance analysis with NeoKISS was therefore beyond the scope of this study. Our main contribution is the description of how microbiological, inflammatory, clinical, and contextual information was associated with original clinical attribution in routine neonatal care.

Most culture-positive episodes in our cohort occurred during the late-onset period and affected very preterm infants, reflecting the patient population of a tertiary NICU with high exposure to invasive procedures and prolonged hospitalization. This pattern is consistent with previous studies showing that late-onset sepsis is concentrated among very preterm and very-low-birth-weight infants and is shaped by device exposure, length of stay, antimicrobial pressure, and local unit ecology [1, 5, 12]. However, because our analysis included only culture-positive episodes and lacked denominators such as admissions, patient-days, and central-line days, these findings should not be interpreted as incidence rates.

The organism spectrum differed clearly between onset periods (Table 1; Fig. 4). Early-onset episodes were relatively uncommon and predominantly involved streptococci and Enterobacterales, consistent with perinatal acquisition. The small number of GBS-positive episodes is in line with contemporary data from high-income settings, in which early-onset GBS disease has declined and the relative contribution of *E. coli*, particularly among preterm infants, has increased [7]. In contrast, late-onset episodes were dominated by healthcare-associated organisms, particularly CoNS, followed by Enterobacterales and *E. faecalis*. This distribution is consistent with neonatal cohort studies identifying CoNS as a leading organism in late-onset bloodstream infection among preterm infants, although the relative distribution of organisms varies between units and over time [5, 6, 9, 13]. Because our late-onset cohort was restricted to infants already hospitalized in the NICU, it primarily represents hospital-onset and intensive care-associated episodes. This may also partly explain the low representation of GBS and limits comparison with studies covering the full spectrum of neonatal late-onset infection.

CoNS-positive episodes represented the largest diagnostic gray zone in our cohort. All 13 CoNS-positive early-onset episodes were attributed as contamination, reflecting the low plausibility of CoNS as vertically acquired invasive pathogens. By contrast, 64 of 101 CoNS-positive late-onset episodes (63.4%) were attributed as infection, illustrating the substantially greater interpretative uncertainty in hospitalized preterm infants with frequent catheter exposure. Similar ambiguity has been reported in other neonatal cohorts [8, 9, 14]. Practical limitations of neonatal care further complicate this distinction. Repeat blood cultures may be difficult to obtain because of restricted blood volume and vascular access, and repeated sampling does not necessarily resolve whether detection of a pathogen represents infection or contamination [15].

Biomarker patterns differed between the early- and late-onset settings. In early-onset episodes, CRP concentrations at sampling showed little separation between attribution groups, whereas maximum CRP was higher in episodes attributed as infection. IL-6, as an earlier inflammatory marker, was already markedly elevated at sampling in the infection-attributed group. This pattern is consistent with the different kinetics of the two biomarkers: IL-6 may rise early during the inflammatory response, whereas CRP frequently increases only after several hours. The findings therefore support the rationale for combining early-phase markers with serial CRP assessment during evaluation of suspected early-onset infection, although the small IL-6 subgroup precludes firm conclusions.

In late-onset episodes, maximum CRP showed the strongest association with original clinical attribution as infection in both univariable and multivariable analyses (Table 3). The same pattern was observed in the CoNS-positive subgroup, suggesting that clinical teams incorporated the inflammatory response as an important part of their clinical decision-making when the organism itself provided limited diagnostic certainty.

Serial CRP measurements are part of routine clinical reassessment. However, since timepoints were not standardized, the measured values of maximum CRP may have been influenced by the clinical course and attribution and thus sampling frequency, introducing incorporation bias.

Nevertheless, CRP alone had limited discriminatory value. Although maximum CRP was substantially higher in episodes attributed as infection, the groups overlapped (Fig. 5). Some episodes attributed as infection showed little or no CRP elevation, whereas some episodes attributed as contamination showed elevated CRP values. This is consistent with the known limitations of CRP in neonatal sepsis diagnostics, including delayed kinetics, limited specificity, and reduced reliability in very preterm infants [16]. In practice, an elevated CRP may increase the apparent plausibility of infection when a blood culture is positive, but it does not prove that the detected organism caused invasive infection.

Other variables provided additional context but were less clearly associated with attribution after adjustment. Shorter time-to-positivity was associated with higher odds of attribution as infection in univariable analysis, but the adjusted estimate was weaker and imprecise. Time-to-positivity may reflect microbial burden and organism-specific growth kinetics, but it is also influenced by blood volume, previous antimicrobial exposure, transport conditions, and the detected species [17]. Similarly, central vascular catheter presence and lower gestational age define a clinical environment in which invasive infection is more plausible, but these factors did not reliably distinguish infection from contamination once a culture-positive episode had occurred.

Detection of the same organism in previous surveillance cultures may also increase the clinical plausibility of invasive infection and is consistent with studies showing that colonization can precede bloodstream infection in NICU populations [4, 18]. However, this association must be interpreted cautiously because surveillance practices differed between organism groups and did not include CoNS. Concordant organism detection may also reflect local microbial ecology or contamination during sampling rather than progression from colonization to invasive infection.

Empirical antimicrobial therapy was initiated at the time of blood culture sampling in most episodes, indicating that initial treatment decisions were generally driven by clinical suspicion before microbiological confirmation.

A strength of this study is its focus on the original clinical attribution of culture-positive episodes over an 11-year period, including separate analyses of early- and late-onset settings and a focused evaluation of CoNS-positive late-onset episodes. This approach captures real-world decision-making that is not fully represented by surveillance definitions alone. Nevertheless, several limitations must be considered. First, the retrospective single-center design limits causal inference and generalizability. Local microbiological ecology, sampling practices, and treatment thresholds may differ from those of other NICUs.

Second, the outcome was the original clinical attribution rather than objectively proven infection status. Thus, misclassification remains possible.

Third, the study included only culture-positive episodes in infants already hospitalized in the NICU. Culture-negative sepsis, culture-negative control episodes, colonized or catheterized infants without suspected infection, and community-onset late infections presenting through the emergency department were not captured. Consequently, the study cannot estimate sensitivity, specificity, predictive values, or comparative infection risk. The absence of admission, patient-day, and central-line-day denominators also precluded calculation of patient- or device-adjusted incidence rates.

Overall, our findings show that interpretation of culture-positive episodes in neonatal intensive care is a process of clinical attribution under uncertainty. The greatest ambiguity occurred in CoNS-positive late-onset episodes, where pathogen plausibility, inflammatory response, time-to-positivity, gestational age, surveillance culture findings, catheter exposure, and clinical evolution could provide incomplete or discordant signals. Prospective studies should capture these variables at predefined clinical decision points and include appropriate comparison groups to develop more reproducible approaches that support both patient safety and antimicrobial stewardship.

## Conclusion

Positive blood culture results in neonatal intensive care do not necessarily indicate clinically relevant bloodstream infection. In this cohort, inflammatory response was most strongly associated with original clinical attribution as infection, while organism identity, time-to-positivity, onset period, and the broader clinical context provided additional but less consistent information. CoNS-positive late-onset episodes represented the greatest diagnostic uncertainty. These findings illustrate how clinicians integrate incomplete and sometimes discordant information when assessing culture-positive episodes. Prospective studies using standardized sampling, predefined clinical decision points, and appropriate comparison groups are needed to develop more reproducible criteria that improve patient outcomes and targeted antimicrobial use in neonatal intensive care.

## Supplementary Information

Below is the link to the electronic supplementary material.

Supplementary Material 1

## Data Availability

The data underlying this study are available from the corresponding author upon reasonable request, subject to applicable data protection and institutional requirements.

## Abbreviations

CoNS: coagulase-negative staphylococci
NICU: Neonatal intensive care unit
OR: Odds ratio
CRP: C-reactive protein
3MRGN: Multidrug-resistant gram-negative bacteria, resistant to three of the four key antibiotic groups
TTP: Time to positivity
