# Supplementary material for: Infection or contamination – Clinical attribution of blood culture-positive episodes in neonatal intensive care: an 11-year retrospective single-center study
Source: BMC Infect Dis. 2026 Oct 15;26:1814. doi: 10.1186/s12879-026-14605-8 (PMC13675962; doi:10.1186/s12879-026-14605-8)
Supplement: Supplementary file 1 [file 12879_2026_14605_MOESM1_ESM.docx]

**Supplementary Table S1. Species-level distribution of organisms detected in culture-positive episodes.** Each species was counted once per culture-positive episode. Polymicrobial episodes contributed more than one organism detection; therefore, column totals may exceed the number of culture-positive episodes. Each organism detection was assigned the onset period and original clinical attribution of the corresponding episode.

| **Organism group** | **Species** | **Overall, n** | **Early-onset** | | **Late-onset** | |
| --- | --- | --- | --- | --- | --- | --- |
|  |  |  | Attributed as infection, n | Attributed as contamination, n | Attributed as infection, n | Attributed as contamination, n |
|  |  | 213 | 12 | 26 | 129 | 48 |
| CoNS | *Staphylococcus auricularis* | 1 | 0 | 0 | 0 | 1 |
|  | *Staphylococcus capitis* | 19 | 0 | 2 | 8 | 11 |
|  | *Staphylococcus epidermidis* | 88 | 0 | 9 | 52 | 27 |
|  | *Staphylococcus haemolyticus* | 15 | 0 | 0 | 10 | 5 |
|  | *Staphylococcus hominis* | 10 | 0 | 4 | 6 | 0 |
|  | *Staphylococcus succinus* | 1 | 0 | 0 | 1 | 0 |
|  | *Staphylococcus warneri* | 1 | 0 | 1 | 0 | 0 |
| Enterobacterales | *Enterobacter cloacae* complex | 8 | 0 | 0 | 8 | 0 |
|  | *Escherichia coli* | 15 | 4 | 0 | 11 | 0 |
|  | *Klebsiella oxytoca* | 2 | 0 | 0 | 2 | 0 |
|  | *Klebsiella oxytoca/Raoultella spp.* | 2 | 0 | 0 | 2 | 0 |
|  | *Klebsiella pneumoniae* | 6 | 1 | 0 | 5 | 0 |
|  | *Pantoea* spp*.* | 1 | 0 | 0 | 1 | 0 |
|  | *Serratia marcescens* | 1 | 0 | 0 | 1 | 0 |
| Streptococcus spp. | *Streptococcus agalactiae* (group B Streptococcus) | 4 | 4 | 0 | 0 | 0 |
|  | *Streptococcus gallolyticus* | 1 | 0 | 0 | 1 | 0 |
|  | *Streptococcus mitis/oralis* | 3 | 1 | 1 | 1 | 0 |
|  | *Streptococcus parasanguinis* | 1 | 0 | 1 | 0 | 0 |
|  | *Streptococcus anginosus group* | 1 | 0 | 1 | 0 | 0 |
| Enterococci | *Enterococcus faecalis* | 11 | 0 | 0 | 10 | 1 |
| Bacillus spp. | *Bacillus cereus* | 6 | 1 | 1 | 2 | 2 |
|  | *Bacillus thuringiensis* | 1 | 1 | 0 | 0 | 0 |
| Environmental organisms | *Micrococcus flavus* | 1 | 0 | 1 | 0 | 0 |
|  | *Micrococcus luteus* | 3 | 0 | 3 | 0 | 0 |
|  | *Roseomonas mucosa* | 1 | 0 | 1 | 0 | 0 |
|  | *Moraxella osloensis* | 1 | 0 | 0 | 0 | 1 |
| Staphylococcus aureus | *Staphylococcus aureus* | 4 | 0 | 0 | 4 | 0 |
| Yeasts | *Candida albicans* | 1 | 0 | 0 | 1 | 0 |
|  | *Candida glabrata* | 1 | 0 | 0 | 1 | 0 |
| Anaerobes | *Bifidobacterium longum* | 1 | 0 | 0 | 1 | 0 |
|  | *Cutibacterium acnes* | 1 | 0 | 1 | 0 | 0 |
| Opportunistic pathogens | *Stenotrophomonas maltophilia* | 1 | 0 | 0 | 1 | 0 |

**Supplementary Table S2. Characteristics of early-onset culture-positive episodes stratified by original clinical attribution.** Values are presented as median (interquartile range) or n (%). Percentages are column percentages based on episodes with available data. IL-6 was available only from 2021 onwards. CoNS, coagulase-negative staphylococci; CRP, C-reactive protein; IL-6, interleukin-6; TTP, time-to-positivity.

| **Feature** | **Overall (n = 35)** | **Attributed as infection (n = 12)** | **Attributed as contamination (n = 23)** | **Missing** |
| --- | --- | --- | --- | --- |
| *Demographic and clinical characteristics* | | | | |
| Gestational age at birth, weeks | 39 (30.5-40) | 33.5 (25-37.2) | 40 (31.5-40.5) | 0 |
| Postnatal age at sampling, days | 1 (0-1) | 0 (0-1) | 1 (0-1.5) | 0 |
| Central vascular catheter present, n (%) | 3 (8.6) | 2 (16.7) | 1 (4.4) | 0 |
| Prior surgery, n (%) | 0 (0) | 0 (0) | 0 (0) | 0 |
| *Microbiological characteristics* | | | | |
| Maternal colonization, n (%) | 6 (17.1) | 6 (50) | 0 (0) | 0 |
| CoNS detection, n (%) | 13 (37.1) | 0 (0) | 13 (56.5) | 0 |
| *Inflammatory markers and time-to-positivity* | | | | |
| CRP at sampling, mg/L | 0 (0-12) | 0 (0-3.25) | 9 (0-17.5) | 2 |
| Maximum CRP, mg/L | 15 (6-44) | 44 (6.5-69.5) | 13 (4.25-24.2) | 2 |
| IL6 at sampling, pg/mL | 309 (63-3461) | 6042 (1816-50000) | 98 (17-350) | 9 |
| TTP, h | 16 (8-34) | 8 (4-10) | 22 (14.5-41) | 0 |

**Supplementary Table S3. Characteristics of CoNS-positive late-onset episodes stratified by original clinical attribution.** Values are presented as median (interquartile range) or n (%). Percentages were calculated within each column using episodes with available data as the denominator. CRP at sampling was available for 64 infection-attributed and 34 contamination-attributed episodes; maximum CRP for 63 and 35 episodes, respectively; and time-to-positivity for 58 and 36 episodes, respectively. Prior colonization with the same organism was not analyzed because surveillance screening for CoNS was not performed systematically. CoNS, coagulase-negative staphylococci; CRP, C-reactive protein; TTP, time-to-positivity.

| **Characteristic** | **Overall (n = 101)** | **Attributed as infection (n = 64)** | **Attributed as contamination (n = 37)** | **Missing** |
| --- | --- | --- | --- | --- |
| *Demographic and clinical characteristics* | | | | |
| Gestational age at birth, weeks | 25 (24-28) | 25 (23.8-28) | 25 (24-27) | 0 |
| Postnatal age at sampling, days | 14 (9-26) | 14 (9-30.2) | 14 (11-20) | 0 |
| Central vascular catheter present, n (%) | 83 (82.2) | 54 (84.4) | 29 (78.4) | 0 |
| Prior surgery, n (%) | 15 (14.9) | 10 (15.6) | 5 (13.5) | 0 |
| *Inflammatory markers and time-to-positivity* | | | | |
| CRP at sampling, mg/L | 6 (0-22.5) | 13 (4.75-28.5) | 0 (0-3.5) | 3 |
| Maximum CRP, mg/L | 22.5 (4-51.8) | 40 (20-72) | 0 (0-4.5) | 3 |
| TTP, h | 16 (10-20) | 15 (7.5-19) | 18.5 (11.5-23.2) | 7 |
